# Recommendations for victim survivability assessment methodology based on the Manchester Arena Bombing Inquiry

**DOI:** 10.1016/j.fsisyn.2025.100634

**Published:** 2025-08-11

**Authors:** Mark Ballard, Anthony M.J. Bull, Jonathan C. Clasper, Alan E. Hepper, Emrys Kirkman, Peter F. Mahoney

**Affiliations:** a Royal Centre for Defence Medicine, Level 2 QEHB, Mindelsohn Way, Edgbaston, Birmingham, B15 2WB, United Kingdom; b Centre for Injury Studies, Imperial College London, Sir Michael Uren Hub, White City Campus, London W12 0BZ, United Kingdom; c Defence Science and Technology Laboratory, Porton Down, Wiltshire, Salisbury, SP4 0JQ, United Kingdom

**Keywords:** Forensic methodology

## Abstract

An expert panel was assembled to report on whether either earlier or different medical treatment could have allowed any of the 22 fatalities of the Manchester Arena Bomb to survive. The aim of this paper is to report on the methodology used by the panel, and to make recommendations for future forensic analyses of explosive events.

The panel comprised individuals with a broad range of expertise who received relevant materials from the Inquiry Solicitors. The methodology used was iterative and comprehensive with the panel meeting together at regular points to: sequentially review all the material; conduct detailed analyses on the material; review the outputs of the analyses; reconcile and address any inconsistencies or points of debate; strictly define the following terms: unsurvivable, unlikely to be survivable, and potentially survivable; and report to the Inquiry. A second level of iteration was overlayed on this due to: early reporting being requested by the Solicitors to the Inquiry prior to all information being provided; and the expert panel conclusions being contested by the family of one of the casualties and the medical experts appointed by their legal teams, resulting in further analyses being conducted by the panel. The following detailed analyses were conducted for each fatality: injury scoring; injury visualisation and mapping; forensic three-dimensional post-mortem CT scan analysis; ballistic injury analysis; blood loss analysis; tourniquet use; blast lung injury; and categorisation and survivability.

The panel concluded that 21 individuals suffered unsurvivable injuries and one suffered potentially survivable injuries. In order to complete their tasking, the panel found that: no one piece of evidence and expertise on its own was sufficient; post-mortem CT imaging to assess anatomical damage and body worn video to analyse physiological response were key; and lack of detailed information on survivors hindered the panel's work.

The clarity of outputs provided by the panel with only the conclusion regarding one of the fatalities being contested highlights the robustness of this panel approach. The following recommendations are made that, in addition to the information that was provided to the panel during this Inquiry, the following information be provided during any future forensic analyses of explosive events: the location of survivors, and uninjured; medical records of the survivors; the three-dimensional plans of the location; structural damage records; and building records and construction technique.

## Highlights

• One individual suffered potentially survivable injuries.
• Twenty one individuals suffered unsurvivable injuries.
• A panel approach provides robust outputs on cause of death and survival likelihood.
• Additional information on survivors would have improved the panel's deliberations.

## 1 Introduction

On the 22 May 2017 an explosive device was detonated in the City Room of the Manchester Arena venue. The response of the Emergency Services to the bombing has been the subject of an Inquiry [1]. The Inquiry report references evidence presented at the hearing and states that the device comprised a combination of high explosive, triacetone triperoxide, surrounded by over 30 kg of around 3,000 small metal items (mainly nuts) [2]. There were 22 fatalities. Greater Manchester Police (GMP) reported that additionally 237 people were physically injured, of whom 91 seriously or very seriously [2].

The Inquiry Chairman asked whether either earlier or different medical treatment could have allowed any of the fatalities to survive. This question could be answered with different levels of certainty for each of the individual fatalities, where “balance of probabilities” leaves significant room for doubt, “beyond reasonable doubt” is a legal standard of proof that sets a higher bar and was used in this case [3].

Survivability after severe trauma is not an exact science and most approaches to this are used to understand survival rate and mortality rate and so cannot be used in a forensic analysis of likelihood of survival for an individual. The consensus-derived Association for the Advancement of Automotive Medicine Abbreviated Injury Scale (AIS) [4] was developed so that the severity scores could be used to give a prediction of survivability, but AIS alone is not suitable in cases with multiple injuries and only scores anatomical injury without reference to physiology [[5], [6], [7]]. Injury scoring based on AIS is frequently used and tools such as the Injury Severity Score [8,9] and New Injury Severity Score [10,11] are used as a way of understanding multi-system trauma. However, the utility of these scores has been questioned in cases of blast [12] and there is evidence that rapidly improving trauma care results in individuals surviving with scores that previously were deemed unsurvivable [13]. In addition to anatomically-based scores, physiological markers can be used to give insight into the condition of the casualty, yet these are affected by pre-existing health conditions, age and physiological resilience. Therefore, a forensic approach must consider all available evidence on anatomical trauma and physiological effects in order to address the question of individual survivability.

There is little literature on how to define survivability, or likelihood of survivability, for an individual and the advances in this field are typically through forensic expert analysis of individual cases that are hard to generalise and which are mostly not available in the open literature. Robust definitions are required as there are examples of casualties deemed ‘unexpected survivors’ [14] meaning that they previously would have been considered unsurvivable, yet due to advanced timely medical interventions did survive.

The Centre for Blast Injury Studies (CBIS) at Imperial College London was instructed by the Inquiry to review evidence and address the question of survivability. The nature of the question meant that there was a need to have experts that understood (and could articulate to a lay audience) the different facets of blast physics, blast effects, blast injury, and the medical treatments and response of the body to blast trauma. CBIS was selected as it is an independent multidisciplinary centre with a reputation for world leading scientific inquiry at the forefront of blast injury research. The Centre could bring in relevant expertise through its extensive network and examine all the strands required for such an investigation. The Centre also had access to people with extensive experience in blast physics, human injury, applied physiology and treatment, along with the facilities to store, handle and analyse the sensitive information that would be required in this examination of the facts. CBIS was not tasked with a review of those who survived the attack. Members of CBIS had assisted with other Inquiries including the 2005 London ‘7/7’ bombing [15] and the reopened Inquest into the 1974 Birmingham pub bombings [15].

The aim of this article is to report on the methodology used by the panel, to discuss key learning points that may be helpful for teams or individuals undertaking similar tasks in the future, and to make recommendations for future forensic analyses of explosive events where survivability is to be considered. The intention is not to cover issues discussed in detail within the publicly available Manchester Arena Reports [16].

## 2 Methods

### 2.1 Materials

A panel was assembled consisting of experts in blast physics, environmental effects of explosions, prevention and mitigation of blast injuries, resuscitation and physiology of blast injury, injury assessment and modelling, cause and treatment of traumatic injuries including surgery, radiology, and post-mortem analysis (Table 1). They comprise the authors of this manuscript.

**Table 1:** Panel members’ specialities and areas of expertise.

Member	Expertise
MB	Medicine (Radiology), Post-mortem Radiology, Military Service, Ballistic and Blast Imaging, Major Incidents, Mass Casualty Events, Expert Witness
AB	Engineering (Biomedical, Biomechanics), Blast Physics, Blast Injury Science including post-mortem analysis, Expert Witness
JC	Medicine (Trauma, Orthopaedics), Surgery, Military Service, Prevention and Mitigation of Blast Injuries, Expert Witness
AH	Engineering (Mechanical), Human Vulnerability, Injury Assessment, Injury Modelling, Personal Protection, Casualty Analysis, Expert Witness
PM	Medicine (Anaesthesia and Critical Care), Resuscitation and Physiology of Blast Injury, Military Service, Humanitarian Service, Forensic Science, Blast and Ballistic Injury, Expert Witness

The panel was provided with relevant materials by the Inquiry Solicitors. An additional expert (EK) provided expertise on physiology and blood loss without being given access to the core solicitor-provided materials, and is co-author of this manuscript.

The materials provided to the panel included witness statements, scene photographs, Body Worn Videos (BWV), Closed Circuit Television (CCTV) images, Disaster Victim Identification (DVI) Images, Post-Mortem photographic images and autopsy reports. Post-Mortem Computer Tomogram (CT) examinations had been carried out to assist the Forensic Pathologists in locating metallic fragments in the casualties and to help direct more focussed examination where needed. These were also provided. Onsite forensic investigation and evidence gathering was undertaken by GMP.

The GMP Operation Manteline team constructed a series of time-line documents for the victims from the CCTV cameras, BWV and mobile phone footage noting who was in attendance (relatives, witnesses, Arena Staff and Emergency Services) and using images to illustrate key events [2].

In the course of their analysis, the panel constructed additional time-line documents to assess the sequelae of injury for specific individuals, where required, to allow understanding of any potential options for medical intervention that may have altered the course of events. These included: rate of blood loss and physiological deterioration and location of anatomical injuries. These were, in turn, compared against witness statements.

Materials were provided on secure encrypted drives and held under conditions of strict control. Documents generated by the CBIS team were password protected and all material underwent disposal at the end of the process in accordance with legal instruction.

## 3 Methodological approach

**Fig. 1:**
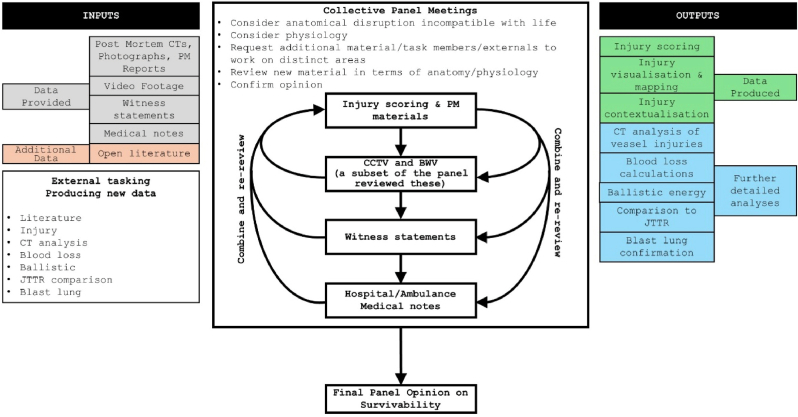
Methodological approach used by the panel.

Our approach is outlined in Fig. 1. All panel meetings took place in person, with all panel members present, except where highlighted in the figure. The material relating to each individual victim was reviewed collectively at panel meetings to understand what had happened to them, the injuries they sustained, and their subsequent time course. Surgical scenario testing was conducted by the panel members collectively to ascertain what the clinical response would be or could be if all information were known at the time of presentation. This information was combined with additional work conducted by individual panel members outside the panel meetings to assess survivability. The primary approach taken during the panel meetings was to sequentially review specific material types first focusing on anatomical disruption, and then focusing on physiology. Anatomical data was derived from post-mortem reports including photographs and CT scans. Physiological data was primarily derived from video footage and witness statements, and, in few cases, medical notes. At all stages the injuries were assessed for consistency and were cross checked with the known explosive type - including specific consideration of primary, secondary, tertiary and quaternary blast effects (Table 2) - and environmental effects (open versus enclosed spaces versus structural collapse) [17].

**Table 2:** Summary of blast injury types as set out by the US Department of Defense [22].

Type	Mechanism
Primary Blast Injury	Overpressure injury resulting from the shockwave interaction with air-containing organs.
Secondary Blast Injury	Injuries produced from fragments. These fragments may be part of the device (primary fragments) or fragments energised from the blast environment (secondary fragments).
Tertiary Blast Injury	Injuries caused by the blast wind and the displacement of the body or parts of the body.
Quaternary Blast Injury	Burns caused by the thermal output of the explosive device.
Quinary Blast Injury	Clinical consequences of “post detonation environmental contaminants” including bacteria, radiation, tissue reactions to fuel, metals, etc.

Casualties could immediately be classified into two groups: those with a clear fatal injury (anatomy based on post-mortem radiology, and other post-mortem documentation including photographs) and those where there were reports of post-incident movement (anatomy plus physiology) or even post-incident intervention (anatomy, physiology, treatment).

This sequential approach was enhanced by a ‘triangulation’ approach where there was cross referencing of all the evidence provided with the other evidence, the scientific and clinical literature, lived experience and expertise (including personal experience of survivors with similar injuries), and analysis including mathematical and computational modelling. Inconsistencies and discrepancies did become apparent and resulted in their resolution or discounting. For example, one witness statement could not have been correct based on all the other evidence provided.

As additional material became available during the course of the instruction (such as further CCTV images and witness statements) these were reviewed and assessed to see if any conclusions needed to be changed.

At all times the panel was chaired to ensure a culture of challenge and to prevent group think.

### 3.1 Post-mortem CT scanning

**Fig. 2:**
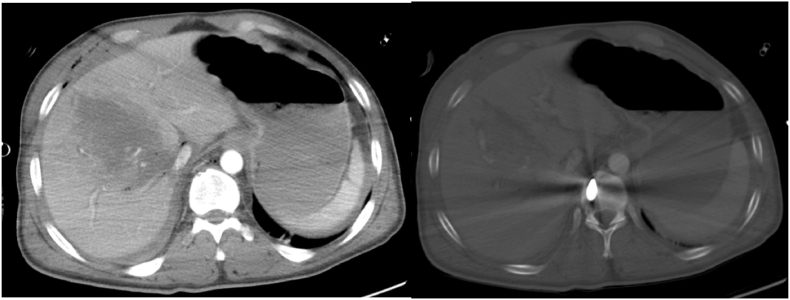
Axial images from a post contrast CT scan of the upper abdomen. Left – soft tissue window. Right – bone window. Fragment penetration through the right side of the abdomen, disrupting the liver. The right lobe shows disruption with haematoma and small locules of gas. Further small locules of gas are seen anterior to the liver in the right upper quadrant within the abdomen and also within the subcutaneous soft tissues of the right upper abdominal wall. Fragment lies in the right of the vertebral body having passed through the liver. Note this is a post contrast CT scan – in the post-mortem setting non-contrasted CT scanning is most commonly performed. This does not represent an actual casualty from the Arena bombing.

The Inquiry Report describes the role of CT scanning [2]. Post-mortem CT scans and forensic post-mortems were performed as soon as possible after the explosion. The acquisition of a multi-slice CT image dataset provides high resolution images of the victim and is especially valuable for the demonstration of foreign bodies and skeletal injury (Fig. 2). The post-mortem CT scanning provided an imaging dataset for the victims of the bomb that was interpreted by consultant radiologists prior to the forensic post-mortem examination. This primary interpretation demonstrated the injury burden and location of foreign bodies (such as bomb fragments and environmental debris) within each victim and identified major injuries – information that facilitated and directed the forensic post-mortem process in concert with X-Ray imaging used during the forensic process to help further target specific fragments. The CT dataset for each victim was then held by the police and released to the panel for re-interrogation as part of the inquiry process.

Retrospective review of the CT images for each victim in concert with the forensic post-mortem report, video evidence and other imagery afforded the panel a greater level of insight into wounding patterns than would have been possible at the time of initial reporting. Multiplanar reformatting of CT scans:

a. demonstrated both complex and subtle fractures and delivered a level of detail that may not be well appreciated in the forensic post-mortem process, and
b. aided appreciation of fragment trajectories through tissue – for example demonstrating injury tracks through tissue in multiple planes, from the skin defect to the retained metallic fragment that caused that defect. Permanent cavities caused by fragments often demonstrate foreign bodies, small locules of gas, or disruption of normal tissue planar anatomy, but the collapsed, temporary, cavity from energy transfer effects are less well appreciated, being ‘filled’ by the collapsed tissue that had been expanded.

In correlation and cross referencing of the post-mortem CT scans with the forensic post-mortem reports and imagery, the panel were able to relate the locations of skin defects, retained fragments and wound tracks to known anatomical landmarks and structures, such as significant blood vessels, methodology that has extensive evidence in the literature [18]. Further correlation of this anatomic detail with the combined still and video imagery and witness statements describing the condition of individuals allowed the panel to infer injury with a high degree of confidence and to assess the likely effects of these injuries on survivability.

In relation to this the Panel were able to:

a. identify injuries that would not normally be found at open post-mortem, for example a minimally displaced acetabular fracture;
b. confidently hypothesise the level of vascular damage that wouldn't be possible with open post-mortem, for example an inferior gluteal artery injury; and
c. assess global fracture patterns, which allowed an assessment of overall energy transfer from bomb fragments, particularly when cavitation was evident (see below).

### 3.2 Injury scoring

The post-mortem reports supported by post-mortem CT imagery, reporting and autopsy photographs were used as evidence to conduct initial Injury Severity Scoring and Internal Wound Mapping. The scoring used was the Association for the Advancement of Automotive Medicine Abbreviated Injury Scale (AIS) [4]. AIS was chosen as it has been widely used for conflict trauma, including related inquiries/inquests [15] and provides consistent scoring across all body regions. Other scoring systems such as the Trauma and Injury Severity Score [19,20] were not considered, because they additionally rely on physiological measures such as the Glasgow Coma Scale [21], systolic blood pressure, and respiratory rate, not available to such a forensic analysis.

Multiple different descriptions are available to apply a score: scoring is not a simple process and should be undertaken by personnel who have been specifically trained by the Association for the Advancement of Automotive Medicine. Such people were available to the panel and the scoring was undertaken by two individuals independent of each other, one from the panel and one not from the panel, and a consensus applied if necessary. This consensus was undertaken in a scoring review meeting between the two individuals to explain and agree on a single score. Very few scores required a consensus and scores differed in description only and not severity. The CT imagery was analysed and reported to the coders by the suitably qualified and experienced Radiologist in the team; however, the coding activity was undertaken independently of the medical expertise on the panel to ensure any collective bias was avoided.

AIS results in six severities which are AIS 1-Minor, AIS 2-Moderate, AIS 3-Serious, AIS 4-Severe, AIS 5-Critical and AIS 6-Maximal. It is worth noting there is not a linear relationship between the injury severities. An AIS 4 is more severe than an AIS 2, but an AIS 4 is not twice the severity of an AIS 2. Also, injuries within the same severity code may not be strictly compatible; some scores are more severe than others within the same severity codes, but they do not justify inclusion in a higher category.

Scores were then recorded in a spreadsheet with the score descriptor and the evidence source to provide traceability. The Injury Severity Score and New Injury Severity Score were calculated and presented alongside Maximum AIS, Number of Injuries and Number of Body regions injured. The scoring was made available to the rest of the panel for the consideration of the injuries of the Manchester Arena attack alongside the other data. During discussions and where clarity was required on the relative severity, AIS scores and justifications were used as reference from the spreadsheet with reference to the original source.

We highlight that the post-mortem photographs additionally give a clear indication of scale. Reporting may have documented the size of a laceration, yet the effect of this on an individual would vary greatly due to their size differentials: from child to adult. Injuries such as blast lung were confirmed from these supplementary sources, and this additional information was used to clarify the original scoring. However, new scores were not added.

### 3.3 Visualisation and mapping tools

In addition to the scoring, two tools were at the disposal of the Defence Science and Technology Laboratory (DSTL) that are used to assist in the design of personal protective equipment:

1. an internal wound visualisation tool to provide an image of internal injuries. This takes the AIS codes and plots them onto an (adult male) image. An example of an image from this tool is shown in Fig. 3; and

**Fig. 3:**
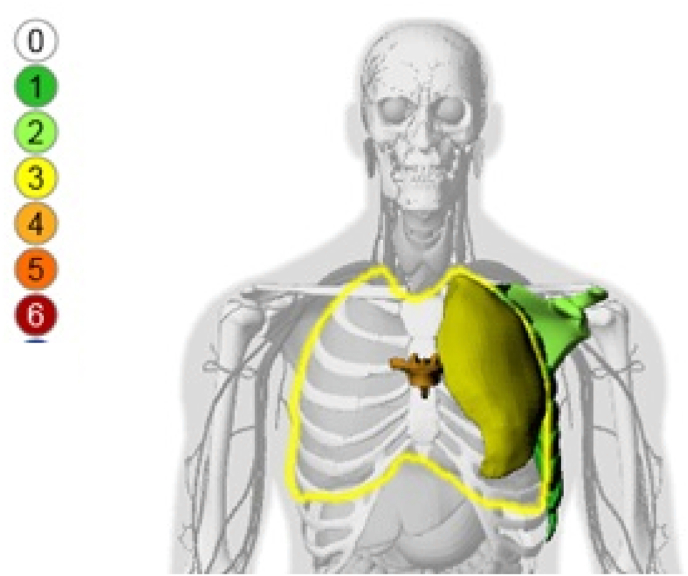
Example image of internal wound mapping – the different colours represent the different AIS severities from zero (no injury) to six (fatal). This does not represent an actual casualty from the Arena bombing.
2. an external wound mapping tool that allows for different types of wounds to be mapped onto an (asexual) representation of a human. This was developed by DSTL. This mapping relies on taking the information from post-mortem images, supplemented by the descriptive text in the post-mortem report. An output from this tool is shown in Fig. 4.

**Fig. 4:**
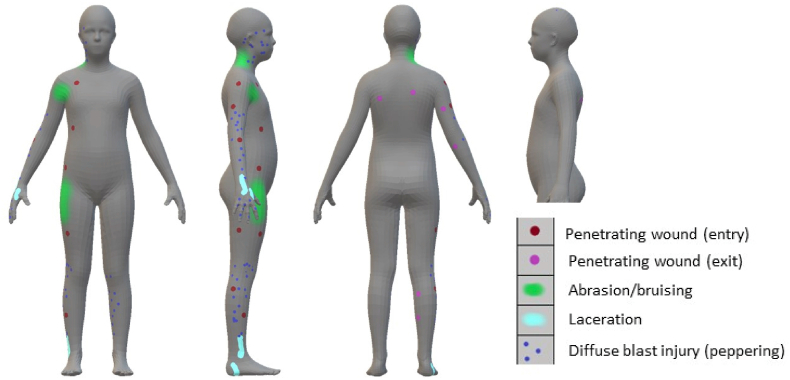
Example image of external wound mapping. This does not represent an actual casualty from the Arena bombing.

These both allow for rapid reference to identify areas of significant trauma, but cannot predict the threat to life of the injuries. The output from these tools were both available to the panel and Inquiry as part of the review process.

### 3.4 Injury categorisation and survivability

Injuries were categorised as being caused by a particular blast injury mechanism as set out by the US Department of Defense [22]. These are summarised in Table 2.

When assessing survivability, the panel used strictly defined wording, noting that none were listed as ‘survivable’ as all victims sustained severe injuries that could have been fatal. The panel definitions are:

*Unsurvivable*: injuries so severe that even if the most comprehensive and advanced medical treatment was initiated, immediately after injury, survival was still deemed impossible [2]. This included either an anatomical injury that is accepted as lethal, or a combination of injuries that in the judgement of the panel would be cumulatively lethal. An example not from the Inquiry would be decapitation.

*Unlikely to be survivable*: injuries so severe that even if the most comprehensive and advanced medical treatment was initiated, immediately after injury, survival would not be expected. We could not, however, deem survival impossible, particularly where there was either conflicting evidence or missing evidence of the time immediately following the explosion. An example not from the Inquiry would be severe burn injury combined with ballistic penetration.

*Potentially survivable:* those injuries that could prove fatal but where there is evidence of, or the authors had direct experience of, individuals who had survived such injuries. This would include anatomical injury that with timely treatment should be survivable, but survival is not guaranteed due to physiological effect and later complications such as sepsis. An example not from the Inquiry would be a cardiac arrest from ventricular fibrillation that didn't receive timely defibrillation.

For each victim a summary was constructed, setting out the most significant injuries, the causative blast mechanism and the panel's view on survivability [2]. An example not from the Inquiry would be: Deceased Person Y; Causative Mechanism: secondary blast; Anatomical Injury: severe penetrating head and chest injury; Survivability: unsurvivable.

The panel were not tasked to analyse injuries suffered by survivors nor comment on care received by survivors. However, the provision of this information would have provided assistance to the panel particularly if there were survivors with an injury burden or with an injury constellation equivalent to the fatalities [23].

### 3.5 Contextualising the primary and secondary blast injuries

The AIS scoring method was originally generated by automotive medicine specialists where relatively low-velocity, but high-momentum, impact is typical. The scoring system has evolved to include blast trauma, but the scoring rules remain, in that the system is reliant upon the injury narrative to justify scores. There was debate during panel meetings whether the high-velocity nature of these injuries were adequately represented in the scoring. This debate did not affect the ascribing of scores, but the panel had combined extensive experience of modelling and treating high-velocity (cavity-causing or ‘cavitating’) projectile wounds (with similarities to secondary blast) which are associated with regions of extensive diffuse injury as well as a direct wound track, that was utilised in the analysis of the anatomical disruption.

To contextualise the nature of the injuries, equations of tissue simulant penetration were used [24] to estimate the energies of a selection of retained fragments. These were estimated as travelling at velocities between 184 m per second and 272 m per second with energies of approximately 200 J to 430 J: as a comparator, the energy of a standard 0.38″ handgun bullet is approximately 227 J (with similar momentum). Device fragments that perforated the body had the potential of travelling at greater velocities and energies, depending on the impact location on the body, but the ability to provide comparative injury was seen as important in the consideration of the total effect of fragment trauma.

The same debate was held with the diffuse and systemic effect of the shockwave from the blast (primary blast) on the person. Recent work has considered the survival rates of military personnel over three different conflicts, in victims subjected to attack from explosive devices [25]. Whilst the nature of these explosive devices over these conflicts were very different, the use of AIS and the New Injury Severity Score served to demonstrate the survival rates of personnel suffering different injuries and how this changed as treatment evolved over time. This work also showed the utility of AIS scoring to give comparison of injury severity in a blast environment.

### 3.6 Findings

The panel presented reasoned evidence that concluded that 21 individuals suffered unsurvivable injuries and one suffered potentially survivable injuries [2]. Two victims were initially assessed as unlikely to be survivable; in both cases there was credible eye witness testimony of post-incident movement and/or post-incident interventions leading the panel to consider that there might have been potential life saving interventions. However, for both of these this was changed to unsurvivable when CCTV and BWV were subsequently made available to the panel to assist with their conclusions. These changes were made, because for one of these victims, this movement was found to be unequivocally impossible based on the BWV. For the second victim, the BWV provided information on “respiratory distress” a result of “lung injury and blood loss” due to “blast lung” and “significant vascular damage” a compounding effect resulting in no possibility of survival (all quotes from Ref. [2]).

The Inquiry accepted that 20 individuals suffered unsurvivable injuries.

The conclusion of unsurvivable injuries was contested by the family of one of these casualties and the medical experts appointed by their legal teams. Further analyses were conducted by the panel to substantiate and test this conclusion. These included:

• further analysis of the full reconstruction of the post-mortem CT image set to re-assess the injuries, to locate, where possible, the major vascular structures, and plot the relation of fractures and ballistic fragments to major vascular structures;
• an assessment of the expected blood loss from these vascular structures which was then correlated with the CCTV and BWV to confirm the injury physiology and compare to the medical literature;
• a calculation of the likely energy delivered to the casualty by ballistic fragments and a comparison with the equivalent from hand-gun bullets [2] (energy equivalent to over 15 handgun bullets);
• a comparison of the combined injuries suffered by this individual with those of a similar severity in a military population and subsequent assessment of the fatality rate in the latter. This was made possible by access to the UK Joint Theatre Trauma Registry (JTTR), a prospectively collected combat trauma registry [26] for whom the casualty's injuries, treatment and survivability was known;
• confirmation of a diagnosis of blast lung from an independent pathologist reviewing post-mortem histology specimens; and
• re-interpretation of the post-mortem CT images by a second radiologist, experienced in post-mortem imaging. This was conducted blind to the prior radiology reports for the victim and concurred with the interpretation of the CT scan.

The victim categorised as potentially survivable had leg injuries which were assessed as treatable by tourniquet. The question then arose as to the window for intervention with tourniquet to allow survival and whether there is a physiological point of no return beyond which intervention cannot help [2]. The panel conducted a review of the clinical literature and reported UK military experience to provide guidance on this. This is considered further in the discussion.

Details of individual casualties and their stories are contained in Saunders [2] as are elements of the conflicting opinions. Some of this needed to be heard in closed court. It is clearly not appropriate to discuss confidential material in this article but the additional detailed methodology to explain the panel's conclusions is presented below. The panel considered the full range of opinions throughout the work.

## 4 Discussion

The complexity of this explosive event and the interpretation of injuries and resulting physiology required input from multiple disciplines, including Bioengineering, Engineering of Blast and Ballistics, Blast Physiology, Military Trauma Surgery, Military Trauma Anaesthesia and Military Radiology.

In addition, panel members had practical experience of managing complex blast and ballistic casualties and contributing to Inquests and major Inquiries. The panel also had a detailed and unique knowledge more extensive than is available in open literature of weapons, weapon functioning and weapon effects. This meant that evidence could be assessed by multiple disciplines and opinion provided to the court grounded in scientific principles, the peer reviewed literature and relevant personal experience. We would suggest that any future requests for such a team use a similar broad-based, expert members process and would recommend the establishment of a register of those with relevant expertise as well as the provision of training.

We found no study in the literature that uses the methodology and approach described here. This is likely due to a difference in tasking where some are descriptive in nature (e.g. Ref. [27]), and others present methodologies for forensic examination (e.g. Ref. [28]) without specifically focusing on the key question of survivability, particularly in the context of the panel here not being responsible for the onsite forensic examination, having been provided this. Yet, these, and others, do recognise the need for a multi-disciplinary approach. Some scoring systems of likelihood of survival have been developed, yet these have not been validated for the types and severities of injuries seen here, and nor do they provide probability of survival scores at the level of confidence we propose here [29,30].

Alternative methodologies such as machine learning assisted trauma analysis [31], forensic injury pattern databases [32], and battlefield injury models [33] can all provide information that benefits forensic analysis and provide statistical analysis of survivability, but none of these can be used – as presented in this paper – to comment with rigour and defensible confidence on potential of survival of individuals.

The Arena Inquiry was very different to the previous analyses the panel had been required to undertake. In the 7/7 Inquiry [15] there was an absence of detailed post-mortem imaging and it was necessary to use computer simulation to assess the blast load experienced by individual casualties [34]. The reopened Birmingham Pub Bombing Inquest relied on witness statements and evidence packs from 1974, and cross referencing this with contemporaneous reports of casualties from the published medical literature [15]. The Arena Inquiry required the review and analysis of multiple CCTV and BWV footage, along with detailed post-mortem images, radiology and reports. This confirms that no single approach can be used, and that the approach needs to be tailored to the individual event.

The CCTV and BWV images provided a window onto the injury physiology and response of individual victims and was used to correlate with witness statements.

Blood pools visible on the images were assessed against elapsed time to estimate how rapidly individuals were losing blood and whether treatment provided was effective, including tourniquet application. This subjective approach seemed to help the Chairman when considering the different witness statements, as well as providing some reassurance to survivors, and also the families of some of the victims. We would recommend that if survival timelines are required, a similar process is utilised.

**Survivability.** The tasking from the Chairman was to comment on survivability. The panel addressed this through strict definition of the following three terms: unsurvivable; unlikely to be survivable; and potentially survivable. This provided clarity to the victims’ families and survivors and we encourage its use in future events. An alternative, and arguably preferable, approach would be for the court to define the levels of survivability to the panel; either approach provides certainty of the standards that are applied.

**Injury scoring.** As AIS injury scoring alone cannot be used to answer questions of survivability, we found it helpful to compare these scores with a database of similarly severely injured who had received advanced medical interventions, noting that our comparator group was for adults in the military setting and so there remain unanswered questions when comparing injuries in the unprotected young.

**Cavitation.** With low energy wounds, for example stabbings, the injury is caused by direct damage to structures that are in the path of the knife; these are readily identified at open post-mortem examination and could be inferred from post-mortem CTs. However, with high-energy wounds, such as in the Arena Bombing, injury can also be caused by cavitation [18,[35], [36], [37]]. The effect of the temporary cavity produced on injury to nearby structures such as major blood vessels is not known in the scientific literature. Post-mortem CT does not detail these secondary effects and therefore it could be proposed that open post-mortem dissection be conducted. We do not recommend this, as post-mortem CT enabled an accurate assessment of blood loss as well as the rate of blood loss, a factor particularly relevant to the Inquiry, where the effect of the timing of medical interventions was of importance. This allowed the panel to confidently state that the injuries of one victim, who was initially conscious, were unsurvivable, regardless of the timing of any medical intervention. In contrast, another had injuries which could have led to survival with earlier, effective application of tourniquets above the site of the identifiable vascular injuries.

**Post-mortem CT.** The availability of post-mortem CT was significant in some cases and added objective, retrospectively reviewable, anatomical data that can be reconstructed in multiple anatomic planes. Although contrast enhancement is not possible in such cases, soft tissue injury and wound tracks were demonstrable, with a specific caveat about vessel injuries adjacent to a wound track. We recommend that post-mortem CT be performed in addition to open post-mortem in blast incidents.

**Blood Loss and Physiological Effects.** Depending on the blood vessel injured and the casualty's heart rate, people can survive vascular injury from seconds to minutes [38]. The complexity of the response is exacerbated by differences in bleeding from veins and arteries [39], and there is no widely agreed methodology to interpret the physiology of inevitable death where the combination of bleeding, reduced oxygen delivery due to lung damage and falling cardiac output is unsurvivable.

**Commercial or improvised tourniquets.** Some casualties of the Arena bombing had improvised tourniquets applied, not all were effective. Lessons learned about improvised tourniquet from prior incidents such as the 2013 Boston marathon bomb attack [40,41] have resulted in a series of recommendations by the American College of Surgeons Committee on Trauma [42], yet there remains a lack of quality standard for such devices.

**Blast Lung.** The panel were asked to comment specifically on the presence or absence of blast lung. The panel found that the classical description of blast lung was unable to shed light on the specific environmental effects, and age-varying responses, that were relevant to the Manchester Arena Bombing. Histological examination in one case was able to confirm blast lung that didn't conform to the classical description. Triangulation of this evidence with witness statements [35] provided confidence in the panel's assessment. The effect of blast lung injury is to further reduce the amount of oxygen available to the organs and tissues and making the overall shock worse and survival less likely when combined with severe bleeding.

There are potential sources of error and variability in this study, including: missing data (for example, the CCTV and BWV footage did not cover all fatalities for all of the time, although in this case this would not have changed the panel's findings); variations in injury reporting (mitigated in this case by having the same two scorers for all fatalities); variations in post mortem reporting (we include a recommendation on addressing this); and alternative methodologies for aspects of the work (including the use of different injury scoring systems).

### 4.1 Recommendations

Based on the work of the panel, and a critical review of all relevant literature, the following recommendations are made:

1. Formalise the development of experts in forensic analysis of casualty survivability in complex blast and ballistic incidents (suggested owner: Home Office through suitable University and Commercial Forensic Providers).
2. Provide guidance and training for the experts and to expand the pool of experts that (suggested owner: Home Office through suitable University and Commercial Forensic Providers):
  a. includes use of the principle of triangulation from multiple sources of evidence;
  b. encourages a multi-disciplinary and cross-disciplinary understanding; and
  c. actively monitors for the risk of group think and the lack of internal (constructive) challenge in the analysis team.
3. Develop a standard form for pathologist reports that is amenable to injury scoring (suggested owner: The Royal College of Pathologists and other national equivalents).
4. Adding post-mortem CT as a routine capability for such complex and large numbers of fatalities cases (suggested owner: national pathologists governing bodies such as The Royal College of Pathologists).
5. When investigating explosive events include data on the injuries suffered by both fatalities and survivors and any treatments delivered (suggested owner: Department of Health and Social Care).
6. Identify specific questions around optimal management of blast casualties that the current literature and guidelines acknowledge are only partially answered, and address these by performing research on (suggested owners: Chief Scientific Officers through research institutes, universities, research funding bodies):
  a. Injury scoring systems to adequately capture the complex injuries associated with blast.
  b. Vessel injuries due to cavitation and secondary fragments (such as from fractured bone).
  c. Detailed computer modelling of the blast, requiring retrospective information on this incident and others including environmental features; this would enable the pre-emptive design of new facilitates to mitigate the effects of potential explosions.
  d. Blast lung injury mechanisms including radiological and histological features in the context of complex loading environments and multiple injury constellations.
7. Increase public training in first aid including commercial and improvised tourniquets (suggested owner: Faculty of Pre-Hospital Care and Voluntary Aid Societies).
8. Agree national definitions of the use of the terms: unsurvivable; unlikely to be survivable; and potentially survivable for victims of blast attacks (suggested owners: Justice System and Department of Health and Social Care).
9. Consider the application of these recommendations to the forensic analysis of all mass casualty events including blast (suggested owners: commissioners of inquiries/inquests, coroners).

## CRediT authorship contribution statement

**Mark Ballard:** Writing – review & editing, Formal analysis, Conceptualization. **Anthony M.J. Bull:** Writing – review & editing, Writing – original draft, Formal analysis, Conceptualization. **Jonathan C. Clasper:** Writing – review & editing, Formal analysis, Conceptualization. **Alan E. Hepper:** Writing – review & editing, Formal analysis, Conceptualization. **Emrys Kirkman:** Writing – review & editing, Formal analysis, Conceptualization. **Peter F. Mahoney:** Writing – review & editing, Writing – original draft, Formal analysis, Conceptualization.

## Relationships

There are no additional relationships related to the subject matter but not directly to this manuscript to disclose.

## Patents and intellectual property

There are no patents related to the work submitted for publication to disclose.

## Other activities

There are no additional activities to disclose.

## Funding

This research did not receive any specific grant from funding agencies in the public, commercial, or not-for-profit sectors.

## Research support

Mark Ballard, Anthony M J Bull, Jonathan C Clasper, Alan E Hepper, Emrys Kirkman, and Peter F Mahoney report financial support was provided by Manchester Arena Inquests.

## Declaration of competing interest

The authors declare that they have no known competing financial interests or personal relationships that could have appeared to influence the work reported in this paper.
